# Health System Decentralization: Creating as Many Problems Than It Solves? Comment on "The Effects of Health Sector Fiscal Decentralisation on Availability, Accessibility, and Utilisation of Healthcare Services: A Panel Data Analysis"

**DOI:** 10.34172/ijhpm.2022.7432

**Published:** 2022-10-18

**Authors:** Dolores Jiménez-Rubio

**Affiliations:** Department of Applied Economics, University of Granada, Granada, Spain.

**Keywords:** Health System Decentralization, Health Outcomes, Healthcare Performance, Regional Inequalities

## Abstract

This comment draws on the study by Rotulo et al about the effects of fiscal decentralization on access, utilization and availability of healthcare resources across Italian regions. We start by discussing the recent trends in health system decentralization worldwide, and then reflect on the rationale and main benefits and the key complexities and challenges of this much debated reform. We address these issues with reference to the recent experience of Italy as well as that of other comparable highly decentralized countries, most notably Spain, paying particular attention to their similarities and contrasts. We conclude that decentralization of health services poses complex challenges and trade-offs which may require careful design of equalisation mechanisms, framework regulation and efficient coordination mechanisms by central and sub central governments.

## Health System Decentralization: A Popular Reform

 Decentralization has been undertaken in many high- and low-income countries in recent decades. For instance, according to recent OECD (Organisation for Economic Co-operation and Development) data available for 22 countries, almost half of them reported a higher level of administrative decentralization in 2018 as compared to 2008, whereas only seven reported a lower score in the decentralization index over the period.^1^ Among the different forms decentralization can take, fiscal decentralization, whereby healthcare spending and taxing responsibilities are transferred to sub national tiers of government, is one of the most prevailing.^2^ There are some temporary exceptions to this increasing trend coinciding with the financial crisis in 2008 and the austerity measures that followed, and the need to take prompt action to coordinate national responses especially during the first wave of the coronavirus disease 2019 (COVID-19) pandemic.^3^ Remarkably, health is the second large spending area for sub national governments across many OECD countries, only after education. Generally, there is a trend towards asymmetric implementation of decentralization reforms within countries as diverse as South Africa, Colombia, France or Italy.^2^ In countries (such as Spain) where this has occurred, investigators can use impact evaluation techniques that take advantage of these “quasi-experiments” to compare outcomes in decentralized regions with regions where decentralization has not yet occurred.^4,5^

## Rationale for Decentralization of Health Services

 Reasons for decentralization are mixed and include improving democracy by promoting local public sector accountability, mostly in developing settings (OECD). Moreover, decentralization is often used in geographically culturally diverse countries as a tool to reduce or alleviate tensions arising due to political, cultural or historical reasons across regions. Notwithstanding these motivations, several studies suggest a clear link between decentralization and relevant economic outcomes such as efficiency, equity, and economic growth. A common argument made for decentralization is that it allows tailoring policies to local needs. For instance, for Spain, a highly decentralised country, and, moreover, regionally very diverse, there is some evidence suggesting that the regions have taken advantage of their degree of autonomy to promote initiatives specifically targeted at addressing heterogenous local preferences and needs. Thus, while some regions with an older than average population have increased beds for long-term patients, in others, efforts have been focused at promoting proximity of healthcare facilities to the local population.^4^ Recent theories on fiscal decentralization argue that subnational governments who finance a larger proportion of their spending with their own sources of revenue are more accountable towards their citizens improving the performance of their jurisdictions.^6^ However, even though a considerable number of studies find a positive association between decentralization and health related outcomes, there are also a few exceptions in the literature, including the findings by Rotulo et al.^7^ On the other hand, a wide consensus exist among researchers on the impact of decentralization on promoting natural experiments and innovation, resulting in new services or initiatives in an effort of regions to politically compete against each other and attract voters. The experience from Germany over the COVID-19 pandemic offers one of the best examples of how flexibility and diversity in local policy making could be highly beneficial in promoting learning by doing.^8^ While requiring people to wear face masks in public places was highly controversial at the beginning of the pandemic, the introduction of compulsory face masks in Jena and other German regions before the rest of the country allowed researchers to exploit this natural experiment and identify mandatory mask wearing as a highly cost effective COVID-19 containment measure which was then implemented countrywide. In Spain, highly autonomous regions have encouraged new management formulas in healthcare and more outsourcing.^9^ This is particularly so in the regions of Catalonia, Valencia and Madrid. Despite the numerous (theoretical) arguments in favour of these public-private collaborations on efficiency grounds, the evidence regarding the superiority of these alternative provider management formulae is inconclusive, basically due to a lack of comparable high quality data. On the other hand, there is some evidence that a recent pioneer reform aimed at increasing freedom of choice of provider (a reform that is being increasingly implemented in many European countries) in the central region of Madrid has played a key role in reducing waiting times to be seen by a specialist.^10^ In addition, decentralized regions in Spain have very often made use of their high level of autonomy and some legal loopholes to bypass national legislation on key aspects. For instance, after the 2012 austerity measures introduced by the central government, which, among other costs containment measures, restricted undocumented immigrants from accessing health services, some of the Autonomous Communities (eg, Andalusia Canary Islands and the Basque Country) decided unilaterally to reintroduce free healthcare for undocumented migrants bypassing the national level regulations.^9^ Finally, an additional positive aspect of health system decentralization regarding flexibility and innovation is that regions, through their own health technology assessment units, have frequently paved the way in the introduction of certain pharmaceutical innovations or technologies. For instance, the Navarre region introduced in 2018 a pilot study for ceasing smoking which was found to be highly successful and cost-effective and while initially was only covered by the regional health system, it was later included in the basic package of the Spanish National Health System.^11^ Likewise, in the United States, a number of policies from unemployment insurance to environmental taxes and regulation, were initially tested in a few states before the federal government decide to adopt similar measures across the country.^2^ Such “laboratory federalism” is argued to improve the efficiency and quality of public policies overall.

## Complexities and Challenges of Health System Decentralization

 On the other hand, decentralization is not without its costs and could bring about challenges of its own as pointed out by Rotulo et al^7^ in the light of theItalian experience. In particular, one of the most common arguments made against decentralization as illustrated in the Italian case is the perpetuation or even exacerbation of regional disparities due to the weaker financial capacity of some regions, especially if decentralisation of financing is involved. This could be the case even if funding for healthcare is distributed equitably, due to differences in local priorities and preferences. Consequently, political pressures for standardisation and equalisation across regions often arise to mitigate inequalities as a result of reforms oriented towards increasing the level of decentralization at the local level. In general, a recurring result in the literature is that decentralization does not have a clear impact on between-regional inequalities but could help to reduce inequalities within regions, although richer regions may generally be in a better position to contain the inequalities.^5^ However, there are a few examples too in the literature of experiences in which decentralization has been detrimental for interregional disparities, such as in the case of Italy where health related outcomes and quality of services are very unequal across regions, and there seems to be an increase in interregional mobility of patients for acute hospital treatment in parallel to decentralization reforms.^7^ The fact that in highly decentralized Spain healthcare related outcomes are found to be less unequal than in Italy might be to a large extent due to the complex system of “compensation funds” that aims at reducing funding imbalance across Spanish regions.^12^ As a result, as opposed to the Italian case, where interregional transfer of patients may be a consequence of important north-south differences in service provision, in Spain, while there is quality driven mobility too, it has remained much the same since 2002.^13^ The issue of much concern for the Spanish decentralization model of healthcare is related to the duplicities and diseconomies of scales that it might bring about, such as an increase in the size and the number of bureaucracies, or the existence of several regional purchasing agencies as opposed to a single one. This could also apply to the hospital sector, where it is argued that in highly devolved and autonomous health services there are far too many barriers (bureaucratic and other) which prevent adequate levels of cooperation and redistribution of patients in those units with higher levels of specialization and expertise.^14^ Interestingly, recent findings based on administrative decentralization measures for a group of OECD countries show that decentralization may promote efficiency up to a point, beyond which there might be overspecialization and fragmentation of services.^1^ Further, an additional drawback of health sector decentralization is the lack of coordination which could be a direct consequence of the devolved nature of health policy, resulting in many cases in unnecessary delays. For instance, after the 2012 austerity measures regarding undocumented immigrants, many regions appealed to the Constitutional Court some of the legal aspects of this legislation, who favoured public provision of healthcare to undocumented immigrants on public health grounds.^9^ The asymmetrical implementation of the law was accompanied by great confusion about the terms of the restrictions not only among the targeted population, but also among doctors and other stakeholders in the system. In addition, more recently, the lack of systematic healthcare data has been pointed out as one of the greatest challenges of the German healthcare system to face the COVID-19 pandemic and a direct consequence of its devolved nature.^15^ Finally, an additional issue of concern of decentralization, which is also present in the Italian decentralization setting,^7^ is the expansion of the private sector within a publicly funded health system, a policy that is permitted by the devolution of competences. While this is not necessarily a disadvantage per se, there is some evidence from Spain of “revolving doors” and lack of transparency associated with the implementation of some public-private mix models of healthcare management.Moreover, although public-private collaborations were very useful in many countries as a way to increase capacity at early stages of the pandemic, the urgency of the measures adopted may have resulted in some cases both in a lack of transparency and inefficiencies.^3^

## Acknowledgements

 The author gratefully acknowledge financial support by the Ministry of Science, Innovation and Universities (grant number PID2019-105688RB-I00).

## Ethical issues

 Not applicable.

## Competing interests

 Author declares that she has no competing interests.

## Author’s contribution

 DJR is the single author of the paper.
